# Environmental *Burkholderia cenocepacia* Strain Enhances Fitness by Serial Passages during Long-Term Chronic Airways Infection in Mice

**DOI:** 10.3390/ijms18112417

**Published:** 2017-11-14

**Authors:** Alessandra Bragonzi, Moira Paroni, Luisa Pirone, Ivan Coladarci, Fiorentina Ascenzioni, Annamaria Bevivino

**Affiliations:** 1 Infections and Cystic Fibrosis Unit, IRCCS San Raffaele Scientific Institute, 20132 Milan, Italy; bragonzi.alessandra@hsr.it (A.B.); moira.paroni@unimi.it (M.P.); 2 Department of Biosciences, University of Milan, 20133 Milan, Italy; 3 Territorial and Production Systems Sustainability Department, ENEA, Italian National Agency for New Technologies, Energy and Sustainable Economic Development, Casaccia Research Center, 00123 Rome, Italy; luisapirone77@gmail.com; 4 Biology and Biotechnology Department “Charles Darwin”, Sapienza University of Rome, 00185 Rome, Italy; ivan.coladarci@gmail.com (I.C.); fiorentina.ascenzioni@uniroma1.it (F.A.)

**Keywords:** *Burkholderia cenocepacia*, mice, environmental, chronic infection, adaptation, lung tissues, *Galleria mellonella*

## Abstract

*Burkholderia cenocepacia* is an important opportunistic pathogen in cystic fibrosis (CF) patients, and has also been isolated from natural environments. In previous work, we explored the virulence and pathogenic potential of environmental *B. cenocepacia* strains and demonstrated that they do not differ from clinical strains in some pathogenic traits. Here, we investigated the ability of the environmental *B. cenocepacia* Mex1 strain, isolated from the maize rhizosphere, to persist and increase its virulence after serial passages in a mouse model of chronic infection. *B. cenocepacia* Mex1 strain, belonging to the *recA lineage* IIIA, was embedded in agar beads and challenged into the lung of C57Bl/6 mice. The mice were sacrificed after 28 days from infection and their lungs were tested for bacterial loads. Agar beads containing the pool of *B. cenocepacia* colonies from the four sequential passages were used to infect the mice. The environmental *B. cenocepacia* strain showed a low incidence of chronic infection after the first passage; after the second, third and fourth passages in mice, its ability to establish chronic infection increased significantly and progressively up to 100%. Colonial morphology analysis and genetic profiling of the Mex1-derived clones recovered after the fourth passage from infected mice revealed that they were indistinguishable from the challenged strain both at phenotypic and genetic level. By testing the virulence of single clones in the *Galleria mellonella* infection model, we found that two Mex1-derived clones significantly increased their pathogenicity compared to the parental Mex1 strain and behaved similarly to the clinical and epidemic *B. cenocepacia* LMG16656^T^. Our findings suggest that serial passages of the environmental *B. cenocepacia* Mex1 strain in mice resulted in an increased ability to determine chronic lung infection and the appearance of clonal variants with increased virulence in non-vertebrate hosts.

## 1. Introduction

Cystic fibrosis (CF) is the most common lethal autosomal recessive disease in Caucasians, with an incidence of approximately 1 in 2500 live births and a prevalence of approximately 100,000 CF patients worldwide [1]. The disease is caused by mutations in the cystic fibrosis transmembrane conductance regulator (*CFTR*) gene, that encodes a chloride channel localized in both secretory and absorbing epithelia. CFTR dysfunction results in abnormal transport of sodium and chloride ions across epithelia affecting the composition of secretions in the lung, gastrointestinal tract, pancreas, liver and other secretory glands. In the airways, CFTR mutations result in a dehydrated viscous mucus that compromises mucociliary clearance and predisposes CF patients to chronic bacterial infections and airway inflammation [2,3]. Life expectancy in CF has improved dramatically in the last few decades, with the median predicted survival age of people born between 2012 and 2016 now 43 years [4]; however, pulmonary infections remain the major cause of morbidity and mortality in people with CF [5].

The CF respiratory tract is a highly diverse and complex ecosystem with a high heterogeneity due to the various environmental conditions [6]. As suggested by Conrad and colleagues [7], resources are limited in the CF lung, and ecologically diverse populations can evolve. Typically, after a period of intermittent colonization of the lung, bacterial infections in CF rapidly become chronic with bacteria persisting until the end of the disease. It is thought that bacterial strain(s) adapt to the CF niche by changing their phenotype(s) and genotype(s) [8,9]; in particular, the persisting pathogens can adapt to disease−specific environmental factors such as anaerobic mucus layers [2], the pressure of the innate immune defence system of the immunocompetent host [3], and aggressive antibiotic therapies administered during the chronic phase of the infection [10]. In accordance with this hypothesis, when newly acquired bacterial strains from the environment are re-isolated after a period of infection in CF lungs, their virulence and pathogenicity differ, as demonstrated for *Pseudomonas aeruginosa* [11]. The adaptation process aims to increase the fitness/survival of bacteria in CF lungs and is generated by the activation of a specific genetic program that can guarantee bacterial genetic variability in stressful conditions [12]. This process ultimately results in therapy resistance, a trait that contributes to the progression of the lung disease, the major cause of morbidity and mortality in CF. To date, bacterial adaptation in CF has been widely described for *P. aeruginosa*; this bacterium appears to undergo a characteristic adaptation process resulting in the production of genetically and phenotypically diverse strains [13]. In this context, there is alarming evidence of an increase in transmissible epidemic strains in major European centers with transmission observed between unrelated CF patients [14,15]. Transmission between patients has been widely documented for *B. cenocepacia* [16], however, our knowledge on the generation of transmissible strains is still lacking. Among the *Burkholderia cepacia* complex (Bcc) species, *B. cenocepacia* is especially problematic in CF patients [16,17,18]. Colonization of the lungs of CF patients by these bacteria is associated with a decrease in long-term survival and, occasionally in a minority of patients, the development of the so-called “cepacia syndrome”, that leads to a frequently fatal acute clinical decline [17]. Several studies have revealed that *B. cenocepacia* is widespread in natural habitats such as the rhizosphere of several crop plants, where it represents one of the predominant Bcc species [19,20,21]. Additionally, it has been reported that *B. cenocepacia* from natural environments are indistinguishable from clinical isolates [22,23] suggesting that humans may acquire Bcc directly from natural environments [21,24]. In support of this hypothesis, candidate determinants related to virulence and transmissibility are not confined solely to clinical *B. cenocepacia* isolates but are also spread among environmental *B. cenocepacia* isolates [25,26,27]. However, understanding of the adaptation process of environmental *B. cenocepacia* strains to the CF airways is still poor [28]; and whether and how *B. cenocepacia* environmental strains adapt to the airways of CF airways remains to be clarified. No data are available on the ability of environmental *B. cenocepacia* strains to adapt to the CF host. Several years ago, Chung and colleagues [29] showed that *B. cenocepacia* strains convert from a nonpersistent to a persistent phenotype in a mouse model of pulmonary infection. In previous work, we explored the virulence and pathogenic potential of environmental *B. cenocepacia* strains and demonstrated that they do not differ from clinical strains in some pathogenic traits showing a similar capacity to maintain a chronic respiratory infection due to the production of similar virulent factors [26]. Furthermore, although environmental strains appear to be less invasive than the clinical ones in polarized CF epithelial cells, they similarly affect epithelia integrity by modulating the presence and distribution of the tight junction protein ZO-1 [27].

In this work, we investigated the host’s role on *B. cenocepacia* adaptation and pathogenicity. We focused our attention on the environmental strain Mex1, belonging to *B. cenocepacia* IIIA. It was collected from the rhizosphere of maize cultivated in a field in Mexico and had previously been characterized for its pathogenicity in vitro and in vivo [26,27]. Notably, in a mouse model of chronic infection, the Mex1 strain caused an extensive inflammatory cell infiltrate in the lung tissues, and has shown a similar capacity to maintain a chronic respiratory infection as the clinical strain LMG16656^T^, probably due to production of similar virulent factors in strains of different origin [26]. In view of the fact that *B. cenocepacia* IIIA, among the Bcc species as well as the other *recA* lineages of *B. cenocepacia* species, is particularly problematic for CF patients, we set up a mouse model of chronic infection to investigate the microbe–host interaction of the environmental strain IIIA. Thus, we carried out serial passages of the environmental *B. cenocepacia* Mex1 in mice, with each round of infection lasting 28 days. Next, we evaluated the ability of the rescued bacteria to adapt to the murine lung tissues and to establish new chronic lung infections. Phenotypic analysis, genetic profiling and virulence of the Mex1-derived clones were also investigated.

## 2. Results

### 2.1. Characteristics of B. cenocepacia Mex1 Strain

The environmental strain Mex1, belonging to *B. cenocepacia* IIIA, was collected from the rhizosphere of maize cultivated in a field in Mexico [26]. Its main characteristics are reported in Table 1. The environmental strain has already shown pathogenic potential in both in vitro and in vivo models [26], a dramatic effect on tight junction integrity and on the presence and distribution of the tight junction protein ZO-1 in CF epithelial monolayers [27]. *B. cenocepacia* Mex 1 strain is also able to form biofilms in nutrient-rich media and can adhere to an abiotic surface as the clinical LMG16656^T^ strain [30,31].

### 2.2. Serial Passages of the Environmental B. cenocepacia Mex1 in Mice

**Figure 1:**
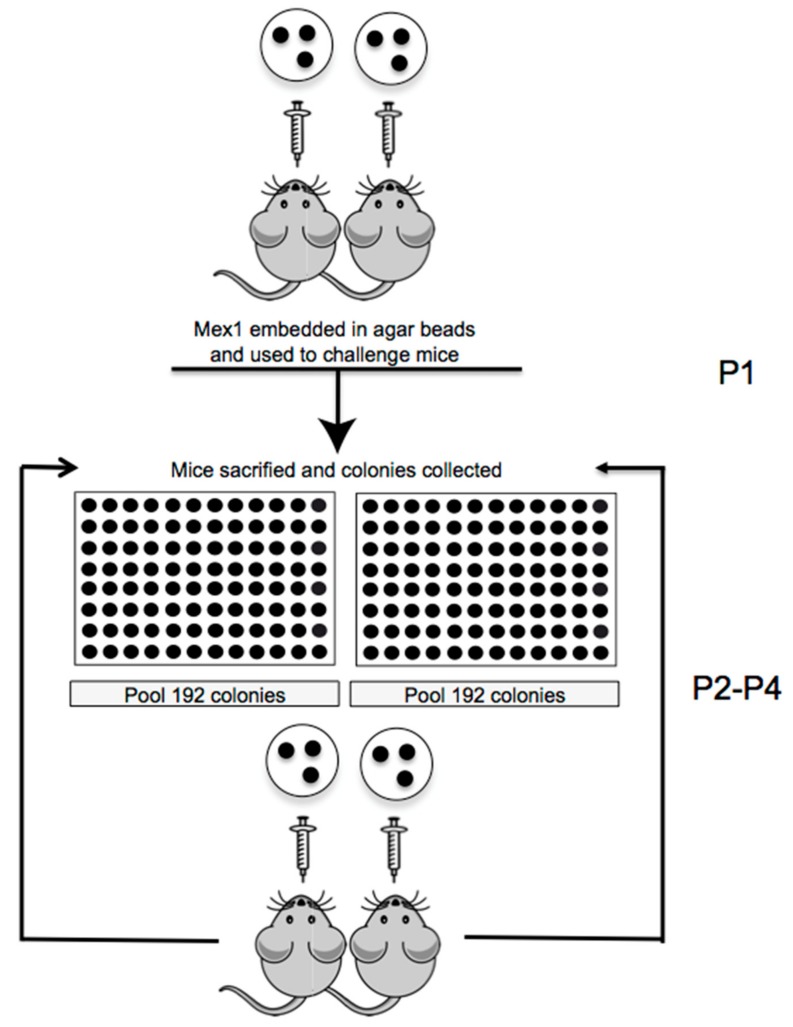
Schedule of sequential chronic *B. cenocepacia* lung infection in mice. Two groups of C57Bl/6NCrlBR mice (*n* = 8–12) were inoculated with 1.5 × 10^7^ CFU/lung of *B. cenocepacia* Mex1 strain embedded in agar beads for 28 days (P1). After 28 days, single colonies were recovered from two groups of infected mice and were re-grown separately; then, they were pulled for two different agar bead preparations and injected in two groups of mice (*n* = 8–9) (P2). The third and the fourth passages in mice (*n* = 8–9) (P3 and P4) were carried out as P2 for 28 days each.

We established sequential chronic infection in C57Bl/6 mice with *B. cenocepacia* Mex1 strain embedded in agar beads. The schedule of experiments is reported in Figure 1. As previously reported, the agar beads mimic the microaerobic/anaerobic environment that allows bacteria to grow in the form of microcolonies and in the mucus of CF patients [2,32]. Sequential infections were established in two groups of C57Bl/6NCrlBR mice challenged with 1.5 × 10^7^ colony-forming units (CFU)/lung and the infection was followed for almost one month (P1) (Figure 1).

**Figure 2:**
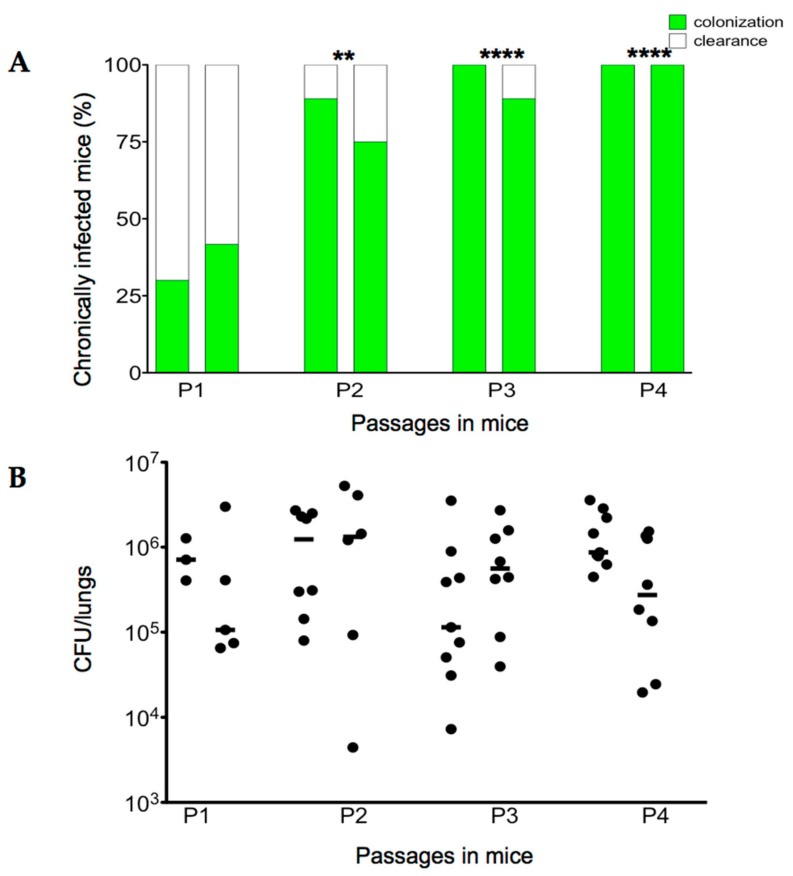
Virulence of *B. cenocepacia* Mex1 after sequential passages in mice. C57Bl/6 mice were infected for four sequential passages by bacteria collected from each passage of 28 days. At each passage mice were sacrificed and evaluated for CFU and percentage of infected mice. (**A**) The percentage of chronically infected mice at different passages. (**B**) The number of CFU of *B. cenocepacia* per lung at different passages. Dots represent individual mice measurements and horizontal lines represent the median values. Two groups of 8–12 mice were analyzed for each passage. Statistical significance is indicated: ** *p* < 0.01, **** *p* < 0.0001.

At 28 days from the first challenge (P1), the incidence of chronic *B. cenocepacia* colonization was 30% in the first group of mice and 41.67% in the second group, with no significant difference between the two groups of mice, confirming previous findings [26,30] (Figure 2A). No mortality was observed and the median value of CFU recovered after 28 days was 7.12 × 10^5^ and 1.06 × 10^5^, in the first and second group of mice, respectively (Figure 2B). For the second passage (P2), 192 single colonies recovered from two infected mice (96 colonies for each mice) were re-grown in vitro separately in a 96-well plate. All 96 colonies were pulled for two different agar bead preparations and injected in two groups of mice, respectively. The same procedure for colony isolation and agar beads preparation was followed for the third and the fourth passages of chronic infection (P3 and P4) (Figure 1).

At the second serial passage in mice (P2), the percentage of infected mice increased significantly to 89% in one group and 75% in the second group (Fisher exact test: *p* = 0.0083 P1 vs. P2). Then, the third and fourth passages (P3 and P4) carried out as described above led to an increase in the percentage of infected mice of between 89% and 100%, respectively (Chi-square Test: *p* = 0.0002 P1 vs. P3; *p* = 0.0001 P1 vs. P3 and P4) (Figure 2A). There was no significant difference between the median value of CFU recovered from the first to the fourth passages in the sub-group of mice maintaining the infection and ranging between 1.14 × 10^5^ and 1.32 × 10^6^ CFU/lung (*p* value = 0.446 P1 vs. P4) (Figure 2B).

### 2.3. Phenotypic and Molecular Characterization of Mex1 Adapted to Murine Lungs

**Figure 3:**
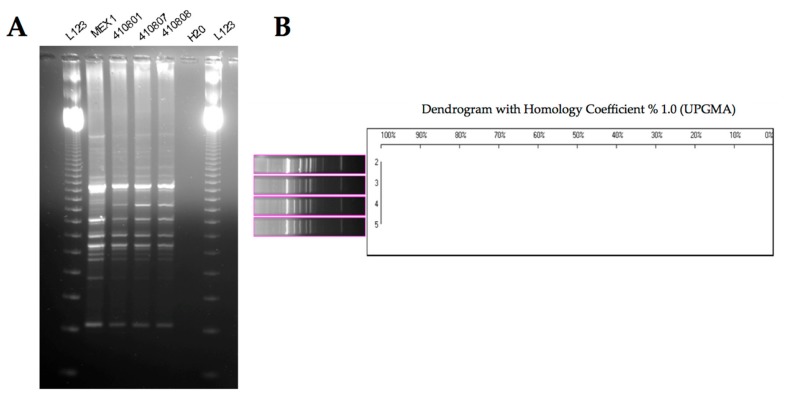
Random amplified polymorphic DNA (RAPD) fingerprints of the *B. cenocepacia* Mex1 challenge and some of its persisting colonies isolated from infected mice. (**A**) The polymorphisms were generated using RAPD primer 270. From left to right: L123, 123-bp molecular size marker ladder; Mex1 strain; clone 410801; clone 410807; clone 410808; negative control. (**B**) The dendrogram showing the clonal relatedness of some persisting colonies, performed with the Unweighted Pair Group Method with Arithmetic mean (UPGMA) by using mathematic averages algorithm programs integral to the Phoretix 1D Pro software. Lanes 2–5: Mex1 strain; clone 410801; clone 410807; clone 41080.

Phenotypic and molecular characterization of bacterial isolates recovered after the fourth passage in mice was carried out, in order to determine whether phenotypic or genetic adaptation occurred. We examined the persistent variants isolated from two groups of eight and nine chronically infected mice after the fourth sequential passages when the percentage of infected mice increased to 100%. Growth on *Burkholderia cepacia* selective agar (BCSA) and genetic profiling by random amplified polymorphic DNA (RAPD) analysis confirmed that the bacteria recovered were genetically indistinguishable from the initially challenged Mex1 strain (Figure 3).

**Figure 4:**
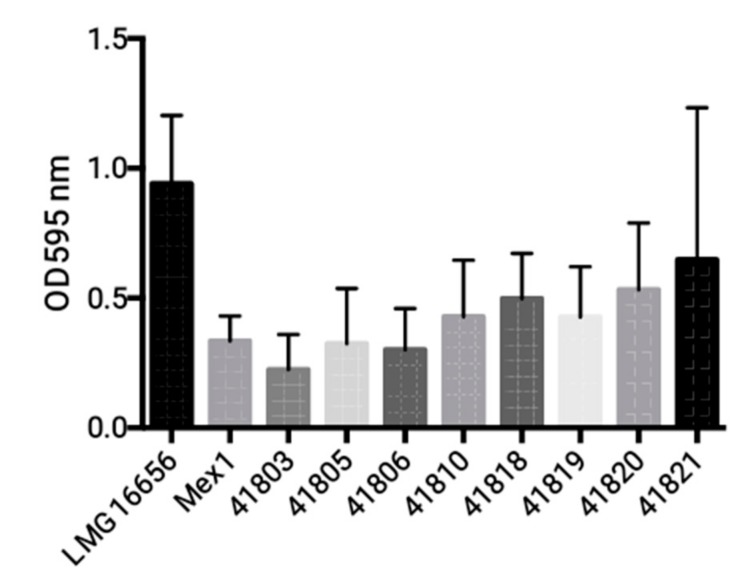
Biofilm formation of Mex1 and some of its derivatives in microtiter plate assay. No significant difference among Mex1 and its derivative clones was found (*p* > 0.05, One-way ANOVA). The clinical LMG16656^T^ formed a higher biofilm in comparison with Mex1 (*p* = 0.0311, Student’s *t*-test). The amount of biofilm was quantified by Crystal Violet staining. Absorbance was measured at 595 nm.

**Figure 5:**
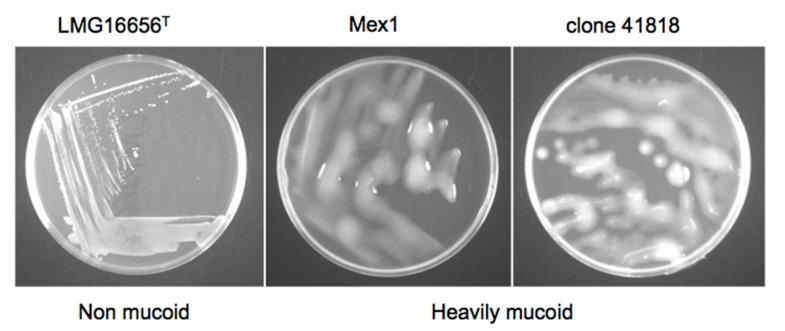
Colonial morphology of Mex1, its derivative (41818 clone as an example) and the clinical *B. cenocepacia* LMG16656^T^ on YEM agar. Strains were grown at 37 °C for 48 h.

*B. cenocepacia* Mex1 strain and its derivatives were finally tested by PCR for the presence of the cable pilin subunit gene (*cblA*), encoding the cable pilus. Both the parent and the Mex1 derivative strains were negative for *cblA* gene, while only the clinical *B. cenocepacia* strain LMG 16656 was found positive (data not shown). No differences in biofilm formation (Figure 4) or colonial morphology (Figure 5) between the parental Mex1 and Mex1-derived clones were observed. Indeed, the mucoid colonial morphology, as assessed on yeast extract mannitol (YEM) agar plates, that correlates with higher levels of EPS production, appeared to be a distinct feature of the Mex1 strains as well as their derivative clones.

### 2.4. Survival and Relative Bacterial Loads of Mex1 and Its Derivative Clones in Infected G. mellonella Larvae

**Figure 6:**
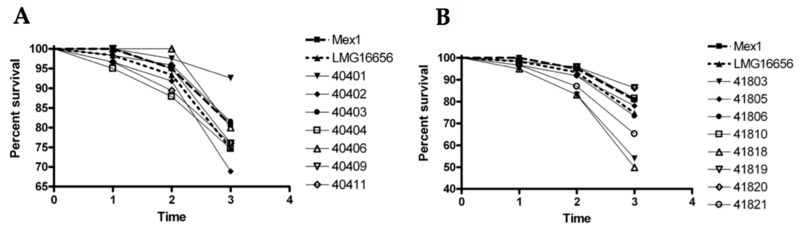
Percent survival of *G. mellonella* following inoculation with Mex1, Mex1-mouse persistent derivatives and the clinical LMG16656^T^. The *B. cenocepacia* strains are indicated on the right: Mex1, thick dashed line; LMG16656, thick dotted line. Y-axis, percent survival of larvae infected with the indicated bacterial strains; X-axis, time post infection (days). Mex1-mouse persistent derivatives were recovered from two groups of eight and 9 C57Bl/6 male mice, and coded as 40 (**A**) and 41 (**B**), respectively.

**Figure 7:**
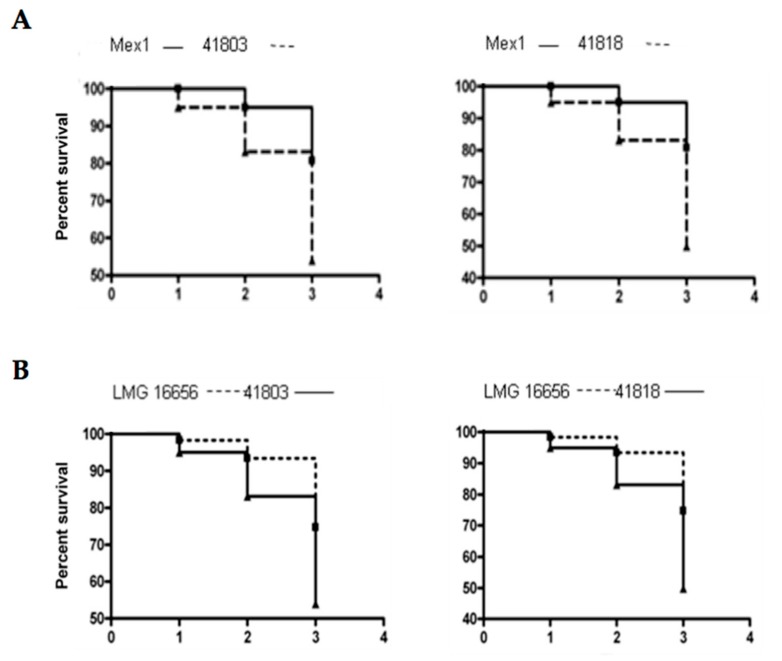
Kaplan-Meier survival plots of larvae injected with the indicated strains. (**A**) The killing ability of Mex1 compared with that of 41803 and 41818 Mex1-derived clones. (**B**) The killing ability of LMG16656 compared with that of 41803 and 41818 Mex1-derived clones. Y-axis, percent survival of larvae; X-axis, time (days) post infection. Statistical analysis was performed by log-rank test.

The *G. mellonella* model is a valuable experimental system for determining the virulence properties of *B. cepacia* complex genetic mutants [33]. Thus, Mex1 and its derivatives recovered after the fourth passage in mice were screened for their ability to kill *G. mellonella* wax moth larvae. The resulting data were analyzed by determining the percentage survival of the infected larvae (Figure 6) and by the Kaplan–Maier method (Figure 7). As expected, the environmental Mex1 strain was significantly less virulent than LMG16656 as assessed by the log-rank test (*p* = 0.0106), demonstrating that the *G. mellonella* assays were suitable to identify differences in the virulence of *B. cenocepacia* strains. Overall, most of the Mex1-derived clones were not significantly different from the parental Mex1 strain, with two exceptions—strains 41803 and 41818—that appeared to be more virulent than Mex1 (Figure 6B).

Accordingly, the Kaplan–Meier survival and the log-rank test showed a significantly higher virulence of these two strains respect to Mex1, with *p* = 0.0156 and *p* = 0.0087 for 41803 and 41818, respectively, while comparison with LMG16656 revealed no significant differences (*p* > 0.05) (Figure 7). Subsequently, the 50% lethal dose (LD_50_) causing 50% death of infected larvae was determined. Results indicated that the two reference strains, Mex1 and LMG16656, showed LD_50_ values equal to 250 and 166, respectively. The Mex1-derivatives were more variable with LD_50_ ranging from 25 (41821) to 500 (41819). The clones 41803 and 41818 were characterized by low LD_50_ values, 59 and 38 respectively.

Overall, we have demonstrated that the Mex1 derivative clones showed a different degree of virulence in comparison with the parental strain. In particular, strains 41803 and 41818 were both significantly more virulent than the parental Mex1 strains, with a virulence degree comparable to that of the clinical LMG16656^T^ strain.

## 3. Discussion

*B. cenocepacia* strains occur naturally in a wide range of environments and are able to persist in human hosts, suggesting that the virulence factors needed to colonize animals and other habitats are similar [34]. Among the natural environments, the rhizosphere is a huge reservoir for bacterial species and a source of human pathogens, due to the enhanced biomass and microbial activity as a result of exudation compounds from the roots [35]. In particular, the maize rhizosphere has a strong influence on the specific host—*B. cenocepacia* interactions, and represents a privileged environment of BCC strains [36,37]. It can also be considered as a reservoir for opportunistic human pathogenic bacteria [24]. Interactions with plant roots might pave the way for bacterial adaptation to mammalian and human cells [35]. In this study, we aimed to investigate whether the rhizosphere *B. cenocepacia* Mex1 strain, that showed a low level of virulence in vivo [26], can increase its fitness in mice establishing niche adaptation through serial passages in a murine model.

Among the genetic lineages of *B. cenocepacia* species, *recA* lineage IIIA appears to have global distribution, predominantly among patients with CF, with high transmissibility and a high mortality rate [23,38,39]. Mex1 constitutes a rare environmental *B. cenocepacia* IIIA strain, isolated from the maize rhizosphere in Mexico [26,27]. Mex1 has a unique sequence type (ST), the ST 423, but genetically it is closely related to internationally spread clones as it belongs to the clonal complex 31, the largest clonal complex of all STs reported in the Multi-Locus Sequence Typing (MLST) *B. cepacia* complex database [16]. Until now, the adaptation process of *B. cenocepacia* to the host environment has been investigated for clinical isolates only [40] and, despite the description of several virulent factors, understanding is still poor [41]. Considering environmental *B. cenocepacia* strains, increased fitness of the *B. cenocepacia* HI2424 strain, belonging to the *recA* lineage IIIB, has been shown suggesting the adaptation of a soil isolate to the onion model [42]. In this case, *B. cenocepacia* adaptation was associated with reduced virulence and a loss of pathogenicity to the nematode *C. elegans*. The large, multireplicon genome and the presence of insertion sequences confer genome plasticity that could explain the versatility of *B. cenocepacia* bacteria and their ability to rapidly adapt to new niches [28]. Environmental strains have defense mechanisms that confer to them a survival advantage in this niche and they have been shown to infect various hosts, including mammals, nematodes and plants [43].

This study revealed that the rhizosphere *B. cenocepacia* Mex1 strain, with a low virulence in mice, increased its ability to cause a chronic lung infection following serial passages in mice that adapt to “local environmental” conditions in the murine lung tissues and establish chronic infections in almost all the infected mice. Our results suggest that chemical–physical characteristics of the host play a role in the selection of virulent bacteria. The phenotypic and genotypic tests we performed did not reveal any differences between strains. Colonial morphology revealed that the phenotype of the Mex1-derived clones recovered from infected mice were indistinguishable from the challenge strain. Our findings suggest that the Mex1 strain and its derivative clones were mucoid, in agreement with results obtained by Zlosnik and colleagues [44], who suggested that the capacity to elaborate EPS may be critical for survival in the environment, the natural niche of *B. cepacia* complex bacteria. Serial passages in mice did not determine any phenotypic switching from to nonmucoid to mucoid forms, as that of clinical and virulent *B. cenocepacia* strains. Also, no differences were observed in the genetic fingerprinting of the parental and the Mex1-derived clones. At any rate, RAPD analysis does not exclude the possibility that point mutations, such as SNPs or other point mutations, may have occurred [45].

When we evaluated the virulence of Mex1-derived clones in the *G. mellonella* larvae infection model, two Mex1-derived clones with a virulence degree more similar to that of the clinical and epidemic LMG16656 strain were found, suggesting adaptation to mice and the acquisition or differential expression of virulence factors. *G. mellonella* is an excellent model for assessing the virulence for a range of microorganisms and provides a rapid and cost-effective alternative for screening a large number of bacteria [46]. The wax moth larvae infection model has recently gained popularity in *Burkholderia* research and has been employed to compare virulence among different BCC species [33]. Recently, it was used to assess the efficacy of the combination therapy (tobramycin with econazole or miconazole) for *B. cenocepacia* [47]. From our results, we can speculate that the ability of *B. cenocepacia* to survive and replicate in various growth niches is due to the high genomic plasticity of this bacterial species and to the expression of host specific virulence factors. When properly regulated, this may help it to compete for survival in different settings [41]. As also suggested by Koskiniemi and colleagues [48], following growth inside a host selection can benefit bacterial mutants through an altered expression of virulence genes that better suit the environment of the host.

## 4. Materials and Methods

### 4.1. Bacteria and Culture Conditions

The bacterial strain used in this study and its properties are shown in Table 1.

### 4.2. Ethical Statement

Animal studies adhered strictly to the Italian Ministry of Health guidelines for the use and care of experimental animals. This study was conducted according to protocols approved by the Institutional Animal Care and Use Committee (IACUC, protocol #369 of 28 July 2008) of the San Raffaele Scientific Institute (Milan, Italy).

### 4.3. Sequential Infection in Mice

Mex1 was included in the agar beads prepared according to the previously described method [26]. Briefly, bacteria were cultured overnight in nutrient broth (NB) at 37 °C to the stationary phase. For agar beads preparation in P2–P4, bacteria isolated from plates were grown in NB broth in a 96-well plate at 37 °C overnight, pooled and centrifuged at 4000 rpm for 10 min. The cells were harvested by centrifugation and re-suspended in 1 mL of PBS (PH 7.4). Bacteria were added to 9 mL of 1.5% NA and subsequently pipetted forcefully into 150 mL of heavy mineral oil, which was kept at 50 °C for bead preparation. The size of the beads was verified microscopically and only those preparations containing beads of 100 µm to 200 µm in diameter were used. The number of *B. cenocepacia* CFU in the beads was determined by plating serial dilutions of the homogenized bacteria-bead suspension on NA plates. The two groups of 8 and 12 C57Bl/6 male mice (Charles River), 6–8 weeks old (20–22 g) were injected with 1.5–2.0 × 10^7^ CFU/lung and the infection was followed for 28 days. Before infection, mice were anesthetized as previously described [30]. After infection, mice were monitored daily for the following clinical signs: coat quality, posture, ambulation, hydration status, and body weight. After 4 weeks, mice were sacrificed, and the lungs were homogenized. Serially diluted lung homogenate samples were plated on NA and the viable cell counts of *B. cenocepacia* were evaluated. Bacterial strains were recovered and the percentage of infected mice was evaluated. Recovery of >1000 CFU from lung cultures was indicative of chronic infection. Next, for the second passage, 192 single colonies recovered after 28 days, from two groups of infected mice were re-grown separately in 96 well plates. Then, they were pooled for two different agar bead preparations and injected in two groups of mice (*n* = 8–9, respectively). Thereafter, third and fourth passages in mice were carried out as described above.

### 4.4. Molecular Characterization of Mex1-Derived Clones

#### 4.4.1. Random Amplified Polymorphic DNA (RAPD) Fingerprinting

The environmental *B. cenocepacia* Mex1 strain and its persisting derivatives recovered from infected mice were genetically typed by random amplified polymorphic DNA (RAPD) analysis, as previously described [49]. Each strain was tested in triplicate, and the entire experiment was repeated three times to verify the RAPD reproducibility. The generated fingerprints were analyzed using the Quantity One software package (Bio-Rad Laboratories, Milan, Italy) and Phoretix 1D PRO software (Phoretix International, Newcastle upon Tyne, UK). The Dice coefficient index was used as similarity measure. The dendrogram was created within Phoretix 1D Pro software by using the unweighted pair group method with arithmetic averages (UPGMA) method.

#### 4.4.2. PCR Amplification of *cblA* Gene

The 664-bp *cblA* DNA coding for the cable pilus was amplified with the primers CBL1 and CBL2, according to the procedure described by Clode and colleagues [50].

### 4.5. Biofilm Formation

Biofilm formation was assessed using the 96-well plate assay and staining the sessile and adherent cells with crystal violet (CV) as described previously [51], with minor modifications [28]. *B. cenocepacia* strains (Mex 1, Mex1-derived clones and the clinical LMG16656^T^) were inoculated from pure cultures grown in M9 minimal medium to mid-exponential (OD_600_ ~ 0.5) phase into at least 6 wells of flat-bottomed 96-well polyvinyl chloride microtiter plates (Greiner Bio-one, Frickenhausen, Germany). Biofilm biomass was quantified by crystal violet staining. Absorbance was measured at 595 nm using a Victor^3^ multilabel counter (Perkin-Elmer, Milan, Italy). Each strain was tested in triplicate, and the entire experiment was repeated three times.

### 4.6. Growth on YEM Yeast Extract-Mannitol (YEM) Agar

Overnight cultures of bacteria were harvested, standardized to an OD590 of 1.0 in PBS, and adjusted to approximately 5 × 10^6^ CFU·mL^−1^. This bacterial suspension was used to inoculate (via a sterile pipette tip) yeast extract medium (YEM; 0.5 g of yeast extract L^−1^ and 4 g of mannitol L^−1^ supplemented with 15 g of agar L^−1^) agar plates. Plates were incubated at 37 °C for 48 h prior to measuring the capacities of isolates to elaborate EPS.

### 4.7. Screening of Mex1-Derived Clones in the G. mellonella Model

The *G. mellonella* infection assay was carried out as described by Uehlinger and colleagues [52] with minor modifications. Overnight bacterial cultures were grown in Luria–Bertani (LB) broth, diluted 1:100 in the same medium and grown to an optical density at 600 nm (OD_600_) of 0.4. Cultures were pelleted, resuspended in 10 mM MgSO_4_ plus 1.2 mg/mL ampicillin and adjusted to OD_600_ = 1. A 5-μL aliquot (approximately 1 × 10^6^ CFU/mL) was injected into the hindmost left proleg. For 50% lethal dose (LD_50_) experiments, a series of 10-fold serial dilutions containing from 10^6^ to 0 bacteria were injected into *G. mellonella* larvae. Ten to fifteen healthy larvae were injected for each strain and incubated in Petri dishes at 30 °C in the dark. The number of live and dead larvae was scored 24, 48 and 72 h after infection; dead larvae were those that did not move in response to touch. All the tests were performed in triplicate. Control larvae were injected with 5 μL of buffer only.

### 4.8. Statistical Analysis

In vivo data were statistically analyzed by using GraphPad Prism. Data analysis was performed using a non-parametric two-tailed Mann–Whitney *U*-test for single comparison for CFU counts. Incidences of chronic colonization were compared using Fisher exact test. Differences were considered statistically significant at *p* values < 0.05.

Biofilm data were analyzed using One-way ANOVA (Prism GraphPad version 6.0 Software Inc., San Diego, CA, USA), considering *p* < 0.05 as the limit of statistical significance.

The Kaplan-Meier curves were used to compare the survival of groups of larvae injected with the *B. cenocepacia* strains. Differences in survival were calculated by using the log-rank test. LD_50_ (CFU) value of each strain was calculated by fitting a linear regression. Survival and LD_50_ were performed using Prism GraphPad version 6.0. A *p*-value of <0.05 was considered to be statistically significant.

## 5. Conclusions

The present study evaluated the ability of the environmental *B. cenocepacia* Mex1 strain, isolated from maize-rhizosphere, to persist and increase its virulence by serial passages during long-term chronic airways infection in mice. In conclusion, we found that the environmental *B. cenocepacia* strain increased its capacity to cause a chronic lung infection after serial passages in mice, adapting to the local environmental conditions of murine lung tissues and establishing chronic infection. However, how environmental bacteria adapt in the complex and variable environment of the host is still largely unknown. Clonal variants with increased virulence in non-vertebrate hosts were found. However, in-depth characterization of Mex1 derivative clones by whole-genome sequencing and comparative bioinformatics analysis is necessary to understand whether intraclonal diversification occurred; this could lead to new targets for future anti-*B. cenocepacia* treatment strategies. Understanding the mechanisms of niche specialization of environmental strains and identifying the common targets of adaptation to the lung environment will allow us to evaluate the potential risk of infection with opportunistic pathogens.

## Figures and Tables

**Table 1:** Characteristics of *B. cenocepacia* Mex1 strain.

Isolate Name	Origin	Sequence Type (ST)	Cci-Encoded Genes	CF and Non-CF Epithelial Cells	Transepithelial Resistance (TER)	References
Mex1	maize rhizosphere (Mexico)	423	positive	low-level invasion	strong disruption of tight junction integrity	[26,27]
